# Effects of Nanosecond Pulsed Laser Cleaning Parameters on the Removal of Thick Paint Coatings from Shipbuilding Steel

**DOI:** 10.3390/ma19112409

**Published:** 2026-06-05

**Authors:** Thongchuea Nutchanat, Satsuki Hiura, Keita Marumoto, Toshitaka Uchida, Takuya Matsuzaki, Motomichi Yamamoto

**Affiliations:** 1 Graduate School of Advanced Science and Engineering, Hiroshima University, Higashi-Hiroshima 739-0046, Japan; 2 Sanwa Dock Co., Ltd., 600 Innoshimashigei-cho, Onomichi City 722-2102, Japan

**Keywords:** laser cleaning, nanosecond pulsed laser, paint removal, thick coating, shipbuilding steel, surface morphology

## Abstract

Thick paint coatings on ship hulls must be periodically removed before repainting; however, conventional abrasive blasting generates secondary waste and poses environmental and occupational health concerns. This study investigates nanosecond pulsed laser cleaning as a non-contact alternative for removing thick marine paint coatings from KE36 shipbuilding steel. A two-layer coating system consisting of anti-fouling and anti-corrosive layers with a total thickness of approximately 1000 μm was examined. The effects of pulse energy, pulse overlap number, and scanning pitch on removal depth, cleaning efficiency, and surface morphology were systematically evaluated. Increasing the pulse energy enhanced coating ablation and enabled complete removal when sufficient heat input density was supplied. A higher pulse overlap number promoted cumulative energy deposition and improved removal depth. Smaller scanning pitches improved spatial overlap between adjacent scan paths and produced more uniform cleaning, whereas excessive pitches caused incomplete removal and periodic surface undulations. The cleaning efficiency approached 100% at a heat input density of approximately 4–5 J/mm^2^. These results indicate that heat input density is a useful process indicator for determining the minimum energy conditions required for full removal of thick coating layers while minimizing thermal effects on the substrate.

## 1. Introduction

Paint coating systems are widely used on ship hull structures to ensure long-term durability and service performance in marine environments. These coatings protect steel substrates from oxidation, corrosion, and biological fouling, which are major causes of material degradation during service [1,2]. However, prolonged exposure to seawater, ultraviolet radiation, mechanical wear, and chemical attack gradually deteriorates the coating layers, resulting in defects such as cracking, peeling, and delamination [3]. Once the coating reaches the end of its service life, surface cleaning and subsequent repainting are required to restore corrosion resistance and maintain the structural reliability of the hull. Conventional surface cleaning processes, including mechanical blasting and chemical treatment, are commonly used in ship repair and maintenance [4]. Among them, abrasive blasting is widely employed because it can remove rust, contaminants, and degraded coating layers with high efficiency [5,6]. Nevertheless, abrasive blasting has several disadvantages, including the generation of large amounts of spent abrasive media, secondary waste, dust, and potential health risks to operators [7,8,9]. These issues are particularly critical in shipyard environments, where large coated areas must be treated and strict environmental regulations are increasingly required. Therefore, alternative surface treatment methods that reduce secondary waste, minimize operator exposure, and preserve the substrate are strongly needed.

Laser cleaning has attracted considerable attention as a promising non-contact and environmentally friendly surface treatment technique [10,11]. Because laser irradiation can be spatially and temporally controlled, it allows selective removal of surface layers with limited mechanical loading on the substrate. In laser paint removal, several mechanisms have been proposed, including thermal ablation, thermal stress, selective vaporization, gasification pressure, light pressure, and plasma-induced effects [12,13]. Among these, photothermal ablation and thermal-stress-induced delamination are particularly important for polymer-based coating removal. Pulsed lasers are effective for such applications because they provide high peak power and localized energy deposition within a short interaction time, enabling efficient ablation while limiting heat diffusion into the underlying substrate [14,15,16]. For thick paint coatings, however, the cleaning process becomes more complex. Coating layers used for marine structures are often composed of multiple functional layers, such as anti-corrosive and anti-fouling coatings, and their total thickness can reach approximately 1000 μm. Complete removal of such thick coatings requires sufficient energy input, but excessive laser irradiation may cause substrate overheating, surface melting, oxidation, or degradation of the surface morphology. Therefore, it is essential to clarify how laser processing parameters affect removal depth, cleaning efficiency, and surface integrity.

In pulsed laser cleaning, pulse energy, pulse overlap number, and scanning pitch are important parameters that determine the spatial and temporal distribution of energy deposited on the coating surface. Pulse energy controls the energy supplied by each pulse and directly affects the local ablation behavior. The pulse overlap number determines the number of pulses applied to the same or adjacent region and therefore influences cumulative energy deposition. The scanning pitch governs the spatial overlap between neighboring scan paths and strongly affects the uniformity of material removal. Although many studies have investigated laser paint removal, systematic understanding of these parameters for high-thickness marine paint coatings remains limited. The scanning strategy is also important for improving cleaning efficiency and suppressing localized heat accumulation. Conventional line scanning can provide high processing rates, but the acceleration and deceleration of galvanometer mirrors near the turning points may increase the local dwell time and cause excessive heat input at the scan edges [17,18,19,20]. In addition, the mechanical inertia of the galvanometer mirror may limit scanning speed and reduce process stability [17,21]. To address these issues, an elliptical scanning path generated using a two-dimensional galvanometer mirror was adopted in this study to improve the spatial distribution of laser irradiation during thick paint removal.

Unlike conventional single-layer coating systems, practical marine coatings generally consist of multilayer structures composed of anti-corrosive and anti-fouling layers with different thermal and optical properties. During nanosecond laser irradiation, these multilayer coatings exhibit more complex laser–material interaction behavior, including non-uniform energy absorption [22,23], cumulative heat accumulation, interlayer removal behavior, and varying ablation characteristics during sequential layer removal. Furthermore, thick multilayer coatings require substantially higher cumulative energy input than thin coatings, increasing the risk of substrate thermal influence during laser cleaning. However, systematic studies on nanosecond pulsed laser cleaning behavior for thick multilayer marine coatings remain limited.

Therefore, the objective of this study was to clarify the effects of nanosecond pulsed laser cleaning parameters on the removal behavior of thick multilayer marine paint coatings applied to KE36 shipbuilding steel. Particular emphasis was placed on identifying suitable combinations of pulse energy, pulse overlap number, and scanning pitch capable of achieving near-complete removal of high-thickness paint layers reaching approximately 1 mm during a single-pass cleaning process. The effects of pulse energy, pulse overlap number, and scanning pitch on removal depth, cleaning efficiency, and surface morphology were experimentally evaluated. Furthermore, heat input density was introduced as an integrated parameter to evaluate cleaning performance and to identify the heat input density required for near-complete coating removal during single-pass cleaning (Number of scans = 1).

## 2. Materials and Methods

### 2.1. Materials

**Figure 1:**
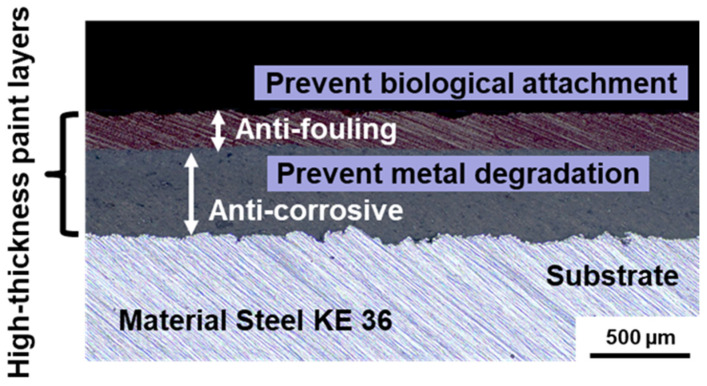
Schematic of the coated KE36 steel specimen consisting of anti-corrosive and anti-fouling coating layers.

Paint-coated KE36 steel was used as the substrate material. KE36 steel is commonly used in shipbuilding applications because of its mechanical properties and suitability for marine structural components. The chemical composition of the steel substrate is listed in Table 1. The coating system consisted of two sequentially applied layers: an anti-corrosive coating and an anti-fouling coating. The total coating thickness was approximately 1000 μm. The anti-corrosive layer (BANNOH 1500R Z, CHUGOKU MARINE PAINTS, LTD., Hiroshima, Japan) was applied to protect the steel substrate from corrosion, whereas the anti-fouling layer (SEAFLO NEO SL Z, CHUGOKU MARINE PAINTS, LTD., Hiroshima, Japan) was applied to suppress biological attachment in marine environments. A schematic cross-section of the coated specimen is shown in Figure 1.

### 2.2. Laser Cleaning System

Laser cleaning experiments were performed using an automated laser processing system consisting of a robotic platform and a nanosecond pulsed laser. A FANUC CRX-20iAL robot was used to control the motion of the laser head relative to the specimen surface. The laser source was a pulsed fiber laser (Laser model: YDFLP-CL-1000-15-W, and Manufacturer: JPT Opto-electronics Co., Ltd., Shenzhen, China) operating at a wavelength of 1064 nm. The maximum average laser power was 1000 W. The beam diameter at the focal position, evaluated from the single-pulse energy distribution, was approximately 460 μm.

**Figure 2:**
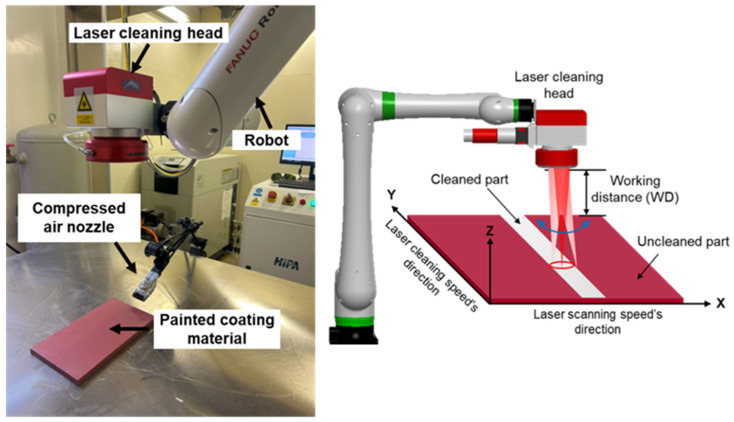
Schematic of the nanosecond pulsed laser cleaning system and elliptical scanning path used for thick paint removal.

The laser beam was scanned using a galvanometer-based optical system. In this study, an elliptical scanning path was employed to distribute the laser energy over the coated surface. The cleaning direction was defined as the X-axis direction, whereas the scanning direction of the laser beam was defined as the Y-axis direction. The scanning pitch was defined as the distance between adjacent elliptical scan paths. A schematic illustration of the laser cleaning system and the scanning path is shown in Figure 2.

### 2.3. Laser Cleaning Parameters

The main laser processing parameters used in this study are listed in Table 2. The average laser power and pulse width were fixed at 750 W and 500 ns, respectively, while the pulse frequency varied from 66 to 220 kHz, resulting in pulse energies ranging from 3.4 to 11.4 mJ. Moreover, the elliptical cleaning beam shape was fixed at 6 mm and 30 mm for the short and long axes, respectively. The scanning pitch and pulse overlap number were varied to adjust the scanning speed and cleaning speed.

The pulse overlap number was used as a derived parameter to quantify the number of laser pulses irradiating a single beam diameter during scanning. It was calculated from the relationship between the beam diameter, scanning speed, and pulse frequency [24]. The distance travelled by the laser spot between two successive pulses is given by $V_{s}/f$. Therefore, the pulse overlap number, n, can be expressed as:

(1) $$n=\frac{D\times f}{V_{s}}$$

where D is the beam diameter, $V_{s}$ is the scanning speed, and f is the pulse frequency.

To evaluate the combined effect of pulse energy, pulse overlap number, and scanning pitch, heat input density was calculated as:

(2) $$HI=\frac{E\times n}{D\times d_{y}}$$

where HI is the heat input density, E is the pulse energy, n is the pulse overlap number, D is the beam diameter, and $d_{y}$ is the scanning pitch.

This definition provides an estimate of the cumulative heat input supplied per unit area during the pulsed laser cleaning process. Unlike the intrinsic single-pulse laser fluence, the present parameter incorporates the effects of repeated pulse irradiation and spatial overlap between adjacent scan paths through the pulse overlap number and scanning pitch. Therefore, it was introduced as an integrated processing parameter for comparing different laser cleaning conditions in terms of cumulative energy deposition behavior.

### 2.4. Surface and Cross-Sectional Characterization

After laser cleaning, the cleaned surfaces were macroscopically observed to evaluate the degree of paint removal and the uniformity of the cleaned area. Surface and cross-sectional morphologies were observed using a digital optical microscope (VHX-S650E, KEYENCE). Cross-sectional specimens were prepared by sectioning the cleaned samples perpendicular to the laser scanning direction and polishing for microstructural observation. For etched microstructural analysis, the polished specimens were chemically etched using a 3% nital solution. In addition, the substrate microstructure beneath the cleaned surface was evaluated by electron backscatter diffraction (EBSD) analysis. For conditions exhibiting non-uniform removal profiles, the coating removal depth was evaluated from cross-sectional images using the average removal depth measured from multiple representative regions along the cleaned cross-section.

Cleaning efficiency was calculated from the ratio between the average removal depth and the initial coating thickness. Surface morphology and cross-sectional features were compared among different laser cleaning conditions to clarify the effects of pulse energy, pulse overlap number, and scanning pitch on material removal behavior.

## 3. Results and Discussion

### 3.1. Effect of Pulse Overlap Number on Removal Depth

The pulse overlap number represents the number of laser pulses applied within a single beam diameter during scanning and therefore determines the degree of repeated energy deposition on the coating surface. Under multi-pulse irradiation, repeated laser exposure increases the cumulative energy input [1,25], resulting in deeper ablation compared with single-pulse irradiation.

**Figure 3:**
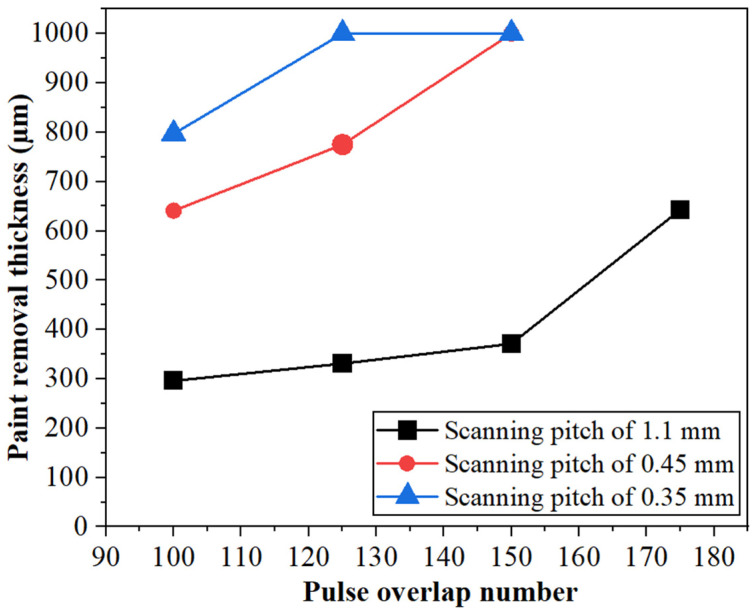
Effect of pulse overlap number on removal depth. The laser power and pulse energy were fixed at 750 W and 6.8 mJ, respectively.

Figure 3 shows the effect of pulse overlap number on paint removal thickness at different scanning pitches under a pulse energy of 6.8 mJ. For all scanning pitch conditions, the paint removal thickness increased with increasing pulse overlap number, indicating that repeated laser irradiation enhanced the laser–coating interaction and promoted progressive material removal. Among the investigated conditions, the scanning pitch of 0.35 mm exhibited the highest removal thickness, reaching approximately 1000 μm at pulse overlap numbers of 125 and 150, corresponding to complete removal of the coating system. Similarly, the scanning pitch of 0.45 mm achieved complete coating removal at a pulse overlap number of 150. In contrast, the scanning pitch of 1.1 mm showed substantially lower removal thickness even at high pulse overlap numbers, indicating insufficient overlap between adjacent scanning tracks and reduced energy accumulation on the coating surface.

However, the increase in removal thickness tended to become less significant once complete coating removal was achieved. This behavior suggests that additional laser irradiation after full coating removal does not further improve cleaning performance and may instead increase unnecessary thermal input to the substrate. Moreover, excessively high pulse overlap numbers can reduce processing efficiency because they increase processing time and energy consumption. Therefore, an appropriate pulse overlap number should be selected to achieve complete coating removal while minimizing excessive thermal loading and maintaining high processing efficiency.

**Figure 4:**
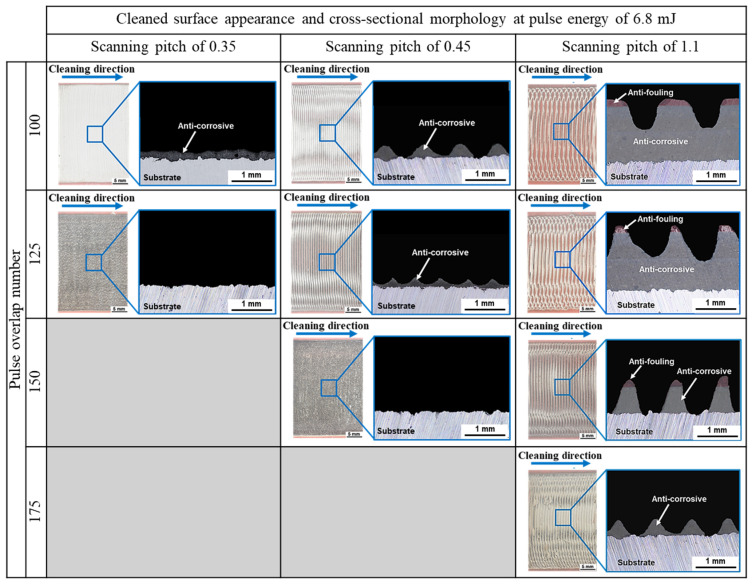
Cross-sectional morphology at different pulse overlap numbers. Optical microscopy observation was performed at 400× magnification.

Figure 4 presents the cleaned surface appearance and cross-sectional morphologies obtained at different pulse overlap numbers and scanning pitches under a pulse energy of 6.8 mJ. At the scanning pitch of 0.35 mm, increasing the pulse overlap number from 100 to 125 promoted effective removal of the anti-corrosive coating layer, and complete coating removal was achieved at a pulse overlap number of 150. A similar tendency was observed at the scanning pitch of 0.45 mm, where residual coating thickness gradually decreased with increasing pulse overlap number, resulting in complete coating removal at a pulse overlap number of 150. In contrast, the scanning pitch of 1.1 mm exhibited incomplete coating removal even at a pulse overlap number of 175, with distinct peak-and-valley morphologies and residual anti-fouling and anti-corrosive coating layers remaining on the substrate surface. These results demonstrate that a smaller scanning pitch combined with sufficient pulse overlap number promotes more uniform energy deposition and stable removal of thick multilayer marine coatings.

### 3.2. Effect of Scanning Pitch on Surface Morphology and Removal Uniformity

**Figure 5:**
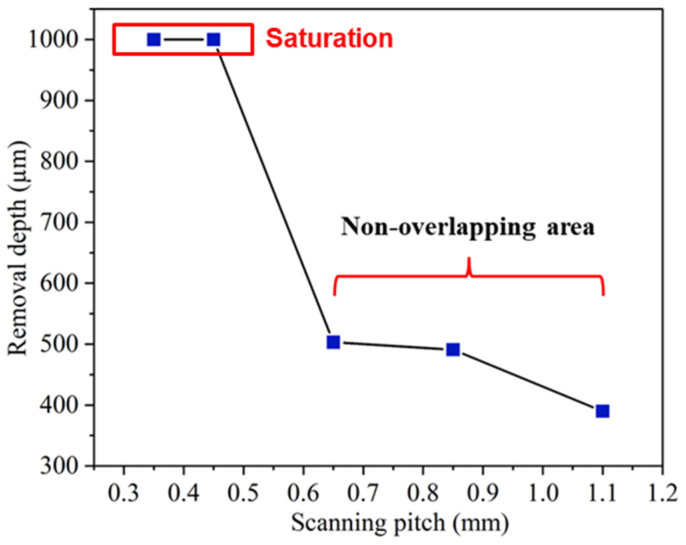
Effect of scanning pitch on removal depth. The laser power, pulse width, pulse energy, and pulse overlap number were fixed at 750 W, 500 ns, 6.8 mJ, and 150, respectively.

In addition to the laser processing parameters, spatial parameters also strongly influence the cleaning behavior. The scanning pitch determines the overlap between adjacent elliptical scan paths and therefore affects the spatial uniformity of energy deposition. Figure 5 shows the effect of scanning pitch on the removal depth. When the scanning pitch was small, particularly 0.35–0.45 mm, the removal depth reached approximately 1000 μm, indicating complete coating removal due to sufficient overlap between adjacent laser paths. As the scanning pitch increased beyond approximately 0.6 mm, the removal depth decreased significantly. At larger pitches, the overlap between adjacent laser paths became insufficient, resulting in discontinuous laser irradiation and incomplete coating removal [26]. In particular, at scanning pitches of 0.85–1.10 mm, the removal depth decreased to approximately 500 μm or less, indicating that only part of the coating thickness was removed.

**Figure 6:**
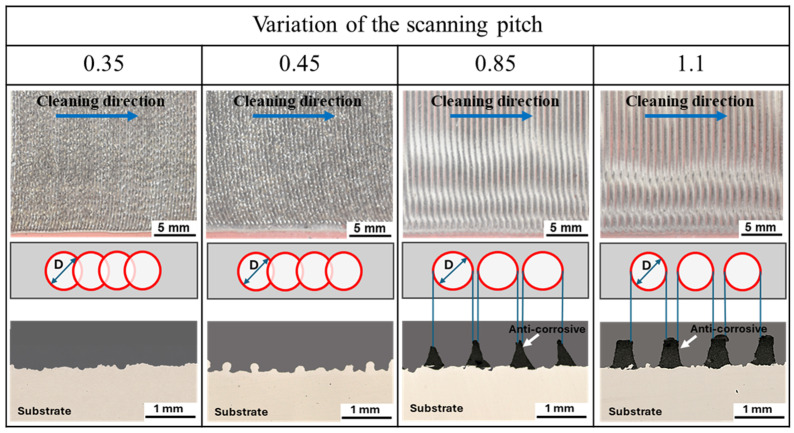
Cleaned surface and cross-sectional morphology at different scanning pitches. Optical microscopy observation was performed at 100× magnification.

The surface and cross-sectional morphologies obtained at different scanning pitches are shown in Figure 6. At scanning pitches of 0.35 and 0.45 mm, the coating was removed relatively uniformly, and the cross-sections showed flat cleaned profiles because the adjacent scan paths sufficiently overlapped. In contrast, larger scanning pitches produced periodic undulations in the cross-sectional profile. These peak-and-valley features correspond to insufficient overlap between adjacent scan paths [1,27]. Because the laser beam has a Gaussian-like spatial energy distribution, the central region of each irradiated spot receives higher energy than the peripheral region. When the scanning pitch is too large, the low-energy peripheral regions are not sufficiently compensated by adjacent paths, resulting in non-uniform material removal and residual coating between neighboring scan tracks.

These results demonstrate that scanning pitch is a critical parameter for achieving uniform coating removal. A small pitch increases spatial overlap and improves cleaning uniformity, but an excessively small pitch may increase heat accumulation and processing time. Under the present conditions, scanning pitches of approximately 0.35–0.45 mm enabled sufficient spatial overlap between adjacent scan paths, resulting in relatively uniform coating removal, whereas larger pitches caused discontinuous irradiation and incomplete removal behavior.

### 3.3. Effect of Pulse Energy on Coating Removal

**Figure 7:**
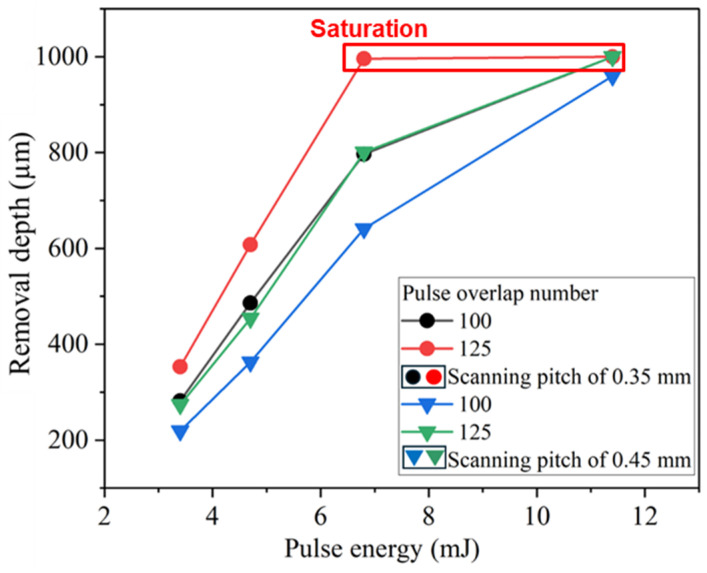
Effect of pulse energy on removal depth. The laser power and pulse width were fixed at 750 W and 500 ns, respectively.

Figure 7 shows the effect of pulse energy on the removal depth under different pulse overlap numbers and scanning pitches. The removal depth increased by increasing pulse energy under all examined conditions. This result indicates that pulse energy is a dominant factor controlling the amount of energy absorbed by the coating per laser pulse. At lower pulse energies, the supplied energy was insufficient to remove the full thickness of the coating, resulting in incomplete cleaning. In contrast, when the pulse energy increased, the supplied laser energy became sufficient to promote more effective coating ablation and material removal.

**Figure 8:**
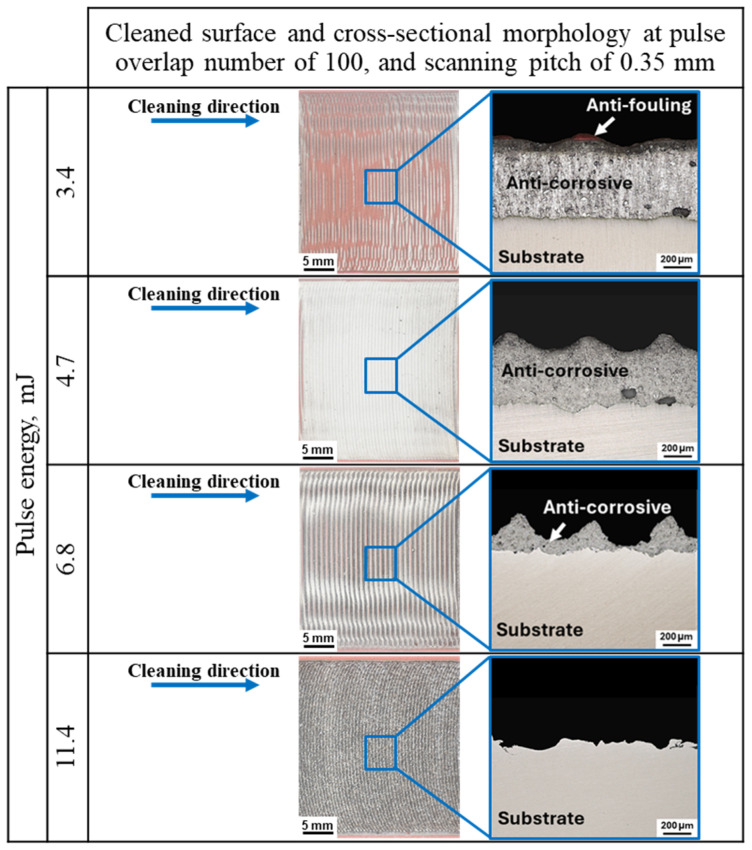
Cleaned surface and cross-sectional morphology at different pulse energies. Optical microscopy observation was performed at 200× magnification.

The surface and cross-sectional morphologies obtained at different pulse energies are shown in Figure 8. At pulse energies of 3.4, 4.7, and 6.8 mJ, residual coating remained on the substrate, indicating incomplete cleaning. The remaining coating layer became thinner as the pulse energy increased, confirming that higher pulse energy promoted deeper ablation. At a pulse energy of 11.4 mJ, substantial coating removal was achieved in a single cleaning cycle, with a removal depth reaching approximately 1000 μm.

The enhanced removal at higher pulse energy can be attributed to increased photothermal ablation, localized vaporization, and thermal-stress-induced coating separation during nanosecond laser irradiation [12,13,14,15,16,28,29]. When the supplied laser energy became sufficiently high to promote effective coating removal, rapid localized heating and thermal expansion occurred within the irradiated region, generating thermal stress at the coating interface and promoting coating delamination and fracture. Correspondingly, the cross-sectional observations in Figure 8 showed a progressive reduction in residual coating thickness and increased ablation depth with increasing pulse energy. These effects collectively enhanced the coating removal performance.

**Figure 9:**
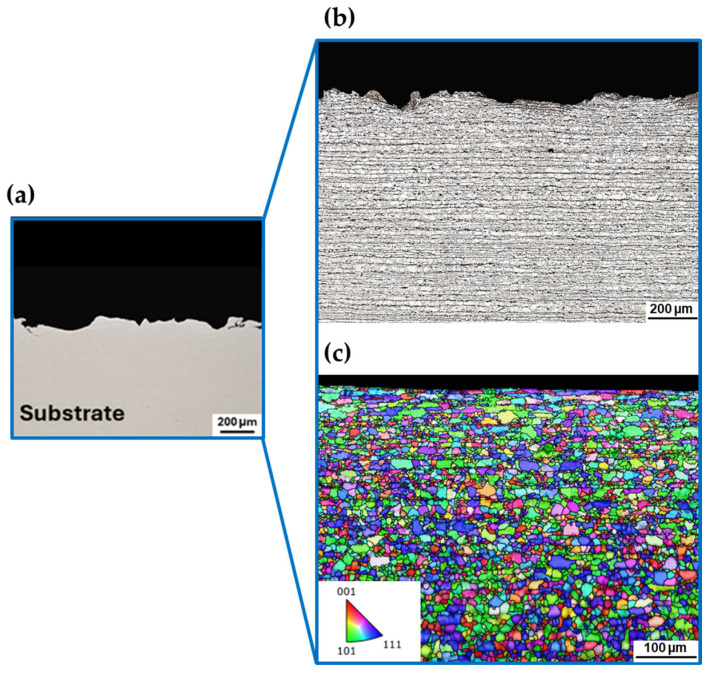
Substrate characterization after complete removal of all coating layers using nanosecond pulsed laser cleaning: (**a**) cross-sectional morphology (200× magnification), (**b**) cross-sectional etched microstructure (200× magnification), and (**c**) EBSD analysis obtained at a pulse energy of 11.4 mJ, pulse overlap number of 100, and scanning pitch of 0.35 mm.

At a pulse energy of 11.4 mJ with a pulse overlap number of 100 and a scanning pitch of 0.35 mm, complete removal of all coating layers was successfully achieved, as confirmed by the cross-sectional morphology shown in Figure 9a. The etched cross-sectional microstructure in Figure 9b revealed no obvious signs of severe thermal damage, such as excessive melting, deep heat-affected regions, or large microcracks within the substrate. In addition, the EBSD analysis presented in Figure 9c showed a relatively uniform grain distribution beneath the processed surface without evident abnormal grain coarsening, indicating that the selected laser parameters enabled effective coating removal while maintaining the integrity of the substrate microstructure. These results suggest that the applied nanosecond pulsed laser conditions provided sufficient energy for complete coating ablation while minimizing detrimental metallurgical alteration to the underlying steel substrate.

### 3.4. Combined Effects of Pulse Overlap Number and Scanning Pitch

**Figure 10:**
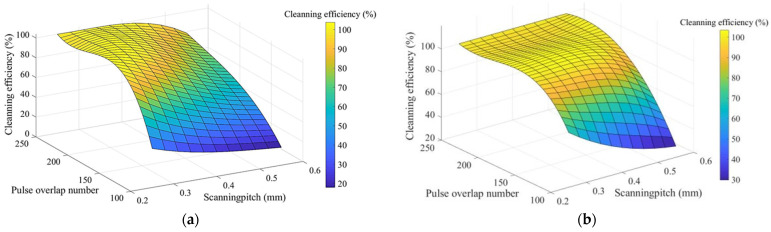
Effect of pulse overlap number and scanning pitch on cleaning efficiency. Three-dimensional surface plots are shown for pulse energies; (**a**) 3.4 mJ; (**b**) 4.7 mJ.

To clarify the combined effects of temporal and spatial energy deposition, the relationship among pulse overlap number, scanning pitch, and cleaning efficiency was evaluated. Table 3 summarizes the experimental conditions used for this analysis. Figure 10 shows three-dimensional surface plots of cleaning efficiency as functions of pulse overlap number and scanning pitch at pulse energies of 3.4 and 4.7 mJ. At both pulse energies, the cleaning efficiency increased with increasing pulse overlap number and decreasing scanning pitch. This result indicates that both temporal overlap and spatial overlap contribute to cumulative energy deposition on the coating surface.

At a low pulse energy of 3.4 mJ, high pulse overlap and small scanning pitch were required to achieve high cleaning efficiency. This is because each individual pulse supplied relatively low energy, and sufficient cumulative energy could only be achieved through repeated irradiation and dense spatial overlap. At a pulse energy of 4.7 mJ, high cleaning efficiency was achieved over a wider range of pulse overlap numbers and scanning pitches. This suggests that increasing the pulse energy reduces the need for excessive pulse overlap or very small scanning pitch.

These results indicate that high cleaning efficiency can be achieved through appropriate combinations of pulse energy, pulse overlap number, and scanning pitch that produce sufficient cumulative heat input density while avoiding excessive irradiation overlap and unnecessary processing time.

### 3.5. Influence of Heat Input Density on Cleaning Efficiency

**Figure 11:**
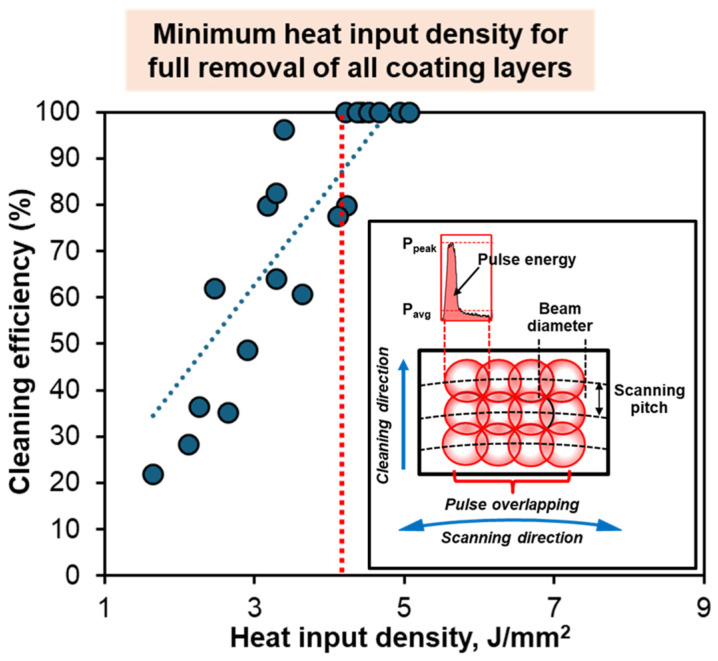
Relationship between heat input density and cleaning efficiency during nanosecond pulsed laser cleaning.

Heat input density was introduced as an integrated processing parameter to evaluate the combined effects of pulse energy, pulse overlap number, and scanning pitch on cumulative energy deposition during laser cleaning [30]. Figure 11 shows the relationship between heat input density and cleaning efficiency during nanosecond pulsed laser cleaning. The results indicate that cleaning efficiency increased with increasing heat input density, reflecting the combined influence of temporal and spatial energy accumulation on coating removal behavior.

At low heat input densities below approximately 3 J/mm^2^, the cleaning efficiency remained below 50%, indicating that the supplied energy was insufficient for effective removal of the approximately 1000 μm thick multilayer coating system. As the heat input density increased, the cleaning efficiency increased rapidly and approached 100% at approximately 4–5 J/mm^2^. This range therefore represents the minimum heat input density required for full removal of all coating layers under the present experimental conditions. Beyond this range, the cleaning efficiency tended to saturate, suggesting that further increases in heat input density provided limited additional improvement in coating removal performance.

The observed trend demonstrates that heat input density is a useful indicator for predicting laser cleaning performance because it incorporates pulse energy, pulse overlap number, beam diameter, and scanning pitch into a single integrated parameter. Therefore, heat input density can be used to identify practical processing conditions for stable and efficient removal of thick multilayer marine coatings. However, excessive energy input may increase substrate heating and surface modification after coating removal.

Compared with previous laser paint removal studies involving thinner coatings or single-layer systems [12,13,14,15,16], the present study required relatively high cumulative energy input because of the approximately 1000 μm thick multilayer marine coating structure. Nevertheless, full removal of all coating layers was achieved under single-pass nanosecond laser cleaning conditions at a heat input density of approximately 4–5 J/mm^2^. These results demonstrate the feasibility of nanosecond pulsed laser cleaning for high-thickness marine coatings and provide a practical processing reference for marine maintenance applications.

Based on the present results, a heat input density of approximately 4–5 J/mm^2^ was sufficient to achieve full removal of all coating layers from KE36 steel under the examined nanosecond pulsed laser cleaning conditions. This value may serve as a practical reference for parameter selection in thick multilayer marine coating removal applications, although further investigation is required for different coating systems, thicknesses, substrate materials, and beam profiles.

Compared with conventional mechanical cleaning methods such as abrasive blasting and grinding, laser cleaning offers several potential advantages for marine coating removal, including non-contact processing, reduced secondary waste generation, high controllability, and selective coating removal capability [7,8,9,10,11]. In addition, nanosecond pulsed laser cleaning enables localized treatment and automated processing using scanning systems. However, removal of thick multilayer marine coatings requires relatively high cumulative heat input and overlapping irradiation, which may increase processing time and energy consumption compared with conventional large-area mechanical cleaning methods. Therefore, although the present results demonstrate the feasibility of nanosecond pulsed laser cleaning for thick marine coating removal, further investigation of processing productivity, operational cost, and large-area cleaning performance is necessary for practical industrial application.

## 4. Conclusions

In this study, nanosecond pulsed laser cleaning was applied to remove thick paint coatings from KE36 shipbuilding steel. The coating system consisted of anti-corrosive and anti-fouling layers with a total thickness of approximately 1000 μm. The effects of pulse energy, pulse overlap number, and scanning pitch on coating removal behavior, cleaning efficiency, and surface morphology were systematically investigated. The main conclusions are as follows.

- Pulse energy strongly affected the removal depth of the thick coating. Increasing the pulse energy enhanced photothermal ablation and thermal-stress-induced coating separation. Full removal of all coating layers with a removal depth of approximately 1000 μm was achieved when the pulse energy was sufficiently high.
- The pulse overlap number controlled cumulative energy deposition during scanning. Increasing the pulse overlap number promoted deeper ablation and improved cleaning efficiency. However, the removal depth tended to saturate once the coating was completely removed, indicating that excessive overlap may increase heat input and processing time without further improving cleaning performance.
- The scanning pitch governed the spatial uniformity of energy deposition. Smaller scanning pitches promoted sufficient overlaps between adjacent elliptical scan paths and produced uniform coating removal. In contrast, larger scanning pitches caused incomplete cleaning and periodic peak-and-valley morphologies due to insufficient spatial overlap.
- The combined effects of pulse energy, pulse overlap number, and scanning pitch were successfully interpreted using heat input density. Under the present conditions, cleaning efficiency approached 100% when the heat input density reached approximately 4–5 J/mm^2^.
- These findings demonstrate that nanosecond pulsed laser cleaning can achieve full removal of thick marine paint coatings without evident severe substrate damage under the examined conditions. heat input density can be used as a practical process indicator for selecting cleaning parameters.

## Figures and Tables

**Table 1:** Chemical composition of KE36 steel substrate.

C	Si	Mn	P	S	Cu	Cr	Ni	Mo	Al	Nb	B	Ti
0.18	0.50	0.90~1.60	0.035	0.035	0.35	0.20	0.40	0.08	0.015	0.02~0.05	0.05~0.10	0.02

**Table 2:** Main laser cleaning parameters used in the experiments.

Laser Parameters	Value	Symbol
Average power, W	750	*P*
Pulse width, ns	500	τ
Pulse frequency, kHz	66–220	*f*
Pulse energy, mJ	3.4–11.4	*E*
Scanning speed, mm/s	121–1012	*V_s_*
Scanning pitch, mm	0.25–1.1	*d_y_*
Cleaning speed, mm/s	1–8	*V_h_*
Beam diameter, µm	460	*D*
Cleaning shape	-	Ellipse
Pulse overlap number	100–250	*n*

**Table 3:** Experimental cleaning parameters used for evaluating combined parameter effects.

Average Power (W)	Pulse Frequency (kHz)	Pulse Width (ns)	Beam Diameter (µm)	Pulse Overlap Number	Pulse Energy (mJ)	Peak Power (W)	Scanning Pitch (mm)	Scanning Speed (mm/s)	Robot Speed (mm/s)
750	66–220	500	460	100–250	3.4–4.7	6.8–22.7	0.25–0.55	200–1000	1–7

## Data Availability

The original contributions presented in this study are included in the article. Further inquiries can be directed to the corresponding author.
